# Integrated transcriptome and metabolome analysis reveals the anthocyanin biosynthesis mechanisms in blueberry (*Vaccinium corymbosum* L.) leaves under different light qualities

**DOI:** 10.3389/fpls.2022.1073332

**Published:** 2022-12-08

**Authors:** Jiaying Zhang, Shuigen Li, Haishan An, Xueying Zhang, Boqiang Zhou

**Affiliations:** ^1^ Forestry and Pomology Research Institute, Shanghai Academy of Agricultural Sciences, Shanghai, China; ^2^ Shanghai Key Lab of Protected Horticultural Technology, Shanghai Academy of Agricultural Sciences, Shanghai, China

**Keywords:** Vaccinium corymbosum L., anthocyanin, light quality, transcriptome, metabolome

## Abstract

**Introduction:**

Blueberry (*Vaccinium corymbosum* L.) is a popular fruit with an abundance of anthocyanins in its leaves and fruits. Light is one of the pivotal environmental elements that affects plant growth and development, but the regulatory mechanism between light quality and anthocyanin formation is poorly understood.

**Methods:**

An integrated transcriptome and metabolome analysis was performed to investigate the effects of white (control), blue (B), red (R), and red/blue (60R/40B) light on blueberry growth and reveal the potential pathway controlling anthocyanin biosynthesis in blueberry leaves.

**Results:**

The anthocyanin content was significantly improved by the blue and red/blue light when compared with white light, whereas there was a significant reduction in the photosynthesis under the blue light, showing an inverse trend to that of anthocyanin accumulation. Transcriptomic analysis resulted in the assembly of 134,709 unigenes. Of these, 22 were differentially expressed genes (DEGs) that participate in the anthocyanin biosynthesis pathway, with the majority being significantly up-regulated under the blue light. Most of the photosynthesis-related genes that were down-regulated were expressed during anthocyanin accumulation. Targeted metabolome profiling identified 44 metabolites associated with anthocyanin biosynthesis. The contents of most of these metabolites were higher under blue light than the other light conditions, which was consistent with the transcriptome results. The integrated transcriptome and metabolome analysis suggested that, under blue light, leucoanthocyanidin dioxygenase (LDOX), O-methyltransferase (OMT), and UDP-glucose flavonoid glucosyltransferase (UFGT) were the most significantly expressed, and they promoted the synthesis of cyanidin (Cy), malvidin (Mv), and pelargonidin (Pg) anthocyanidins, respectively. The expression levels of dihydroflavonol 4-reductase (DFR) and OMT, as well as the accumulation of delphinidin (Dp), peonidin (Pn), and petunidin (Pt), were significantly increased by the red/blue light.

**Discussion:**

The blue and red/blue lights promoted anthocyanin biosynthesis via inducing the expression of key structural genes and accumulation of metabolites involved in anthocyanin synthesis pathway. Moreover, there was a possible feedback regulating correlation between anthocyanin biosynthesis and photosynthesis under different light qualities in blueberry leaves. This study would provide a theoretical basis for elucidating the underlying regulatory mechanism of anthocyanin biosynthesis of V. corymbosum.

## Introduction

Blueberry (*Vaccinium corymbosum* L.) is a fruit-bearing shrub in the genus *Vaccinium*, which is part of the heath family (Ericaceae). It is a commercially important small fruit crop owing to its healthy and flavorful bioactive compounds, including vitamins, anthocyanins, and other phenolic compounds (Zhang L. et al., 2019; Kalt et al., 2020). Blueberries are inherently high in anthocyanins and can be used to fight some human cancers (Seeram et al., 2006) and regulate oxidative stress in organs (Song et al., 2016), blood glucose levels (Fallah et al., 2020a), and inflammatory responses (Fallah et al., 2020b). Due to these potential health benefits, interest in blueberry anthocyanins has been increasing (Chai et al., 2021). The previous studies on anthocyanin biosynthesis have mainly focused on blueberry fruits and discovered some crucial differentially expressed genes or transcription factors related to anthocyanin biosynthesis pathway (Lin et al., 2018), but reports on anthocyanin accumulation in the leaves have been limited. Leaves are abundant in metabolites and the site of secondary plant metabolite production. Anthocyanins are one of the most important classes of secondary metabolite, and the anthocyanins in leaves may promote plant growth and development (Guo et al., 2020). Secondary metabolites, including flavonoids and anthocyanins, extracted from *Lithocarpuspo lystachyus Rehd* and blueberry leaves, can play a crucial role in the treatment of diabetes, hypertension, and chronic illnesses (Ehlenfeldt and Prior, 2001; Takeshita et al., 2009; Hou et al., 2011; Hou et al., 2012; Li et al., 2021). Anthocyanins in blueberry leaves may also be involved in the scavenging of reactive oxygen species (ROS), which could enhance disease resistance in humans (Sakaida et al., 2007). Additionally, the blueberry leaf is a commonly used drug for treating human thrombotic stroke in clinical trials (Nagao et al., 2008). Nevertheless, the true value of the blueberry plant including its leaves has not been fully explored yet, resulting in the unnecessary waste of this resource. Exploring and studying nutritious compounds, such as the anthocyanins in blueberry leaves, will lead to more efficient utilization of fruit resources and help to satisfy the growing demand for natural foods and medicines. Therefore, there is substantial interest in understanding the anthocyanin metabolism in blueberry leaves. Regrettably, our current understanding of the molecular mechanisms underlying anthocyanin biosynthesis is limited and how environmental factors regulate anthocyanin accumulation in blueberry leaves is unclear.

Anthocyanins are the most conspicuous class of flavonoid metabolic branches involved in the phenylpropane metabolic pathway, which presents numerous secondary metabolic pathways in plants (Allan et al., 2008; Wang et al., 2017). The anthocyanin biosynthesis pathway in plants is well understood and key enzyme genes involved in anthocyanin biosynthesis, including cinnamate 4-hydroxylase (*C4H*), 4-coumarate-CoA ligase (*4CL*), chalcone isomerase (*CHI*), chalcone synthase (*CHS*), flavanone 3-hydroxylase (*F3H*), flavonoid 3′-monooxygenase (*F3’H*), flavonoid 3′, 5′-hydroxylase (*F3’5H*), dihydroflavonol-4-reductase (*DFR*), phenylalanine ammonialyase (*PAL*), anthocyanidin synthase (*ANS*), and UDP-glucose flavonoid glucosyltransferase (*UFGT*), have been identified in the colored tissues of several plants. Moreover, the pathway is regulated by the interaction of DNA-binding R2R3MYB transcription factors and MYC-like basic helix–loop–helix (bHLH) and WD40-repeat proteins, i.e., MYB-bHLH-WD40 transcription factor complexes (Jaakola, 2013). The up-regulation of these genes could promote anthocyanin accumulation (Liu et al., 2016; Gao et al., 2021). Aside from these genes and transcription factors, anthocyanin biosynthesis is also influenced by environmental factors (Liu et al., 2016; Lin et al., 2018). A considerable amount of new research has focused on elucidating the environmental regulations controlling anthocyanin biosynthesis, specifically the impact of light-mediated regulation (Jaakola, 2013). Light exposure can increase anthocyanin concentrations, especially in the fruit skin, and the shading of fruits can have the opposite effect (Takos et al., 2006). In the pathway of light-controlled anthocyanin biosynthesis, light-receptor could interact with the CONSTITUTIVE PHOTOMORPHOGENIC1 (COP1), which regulated the expression level of the ELONGATED HYPOCOTYL5 (HY5), or interact directly with certain anthocyanin biosynthesis-related MYB transcription factors to promote the transcription of structural pathway genes regulating anthocyanin biosynthesis (Stracke et al., 2010; Li et al., 2012). In recent decades, researchers have found that light quality can significantly affect the biosynthesis of anthocyanins; UV and other types of light (e.g., blue light) have been associated with the regulation of anthocyanin biosynthesis (Ordidge et al., 2011; Li et al., 2013). Additionally, light quality was found to regulate the gene expression patterns related to anthocyanin synthesis and the regulation of anthocyanin accumulation and stability (Briggs and Olney, 2001; Zoratti et al., 2014). During anthocyanin biosynthesis, the regulatory effects of anthocyanin pigmentation were found to vary according to the different light quality, which influences the levels of key enzymes that are involved in anthocyanin biosynthesis (Mol et al., 1996). For most plants, blue light induces an increase in the transcript levels for the *PAL1*, *CHS*, *CHI*, and *DFR* genes, which encode anthocyanin biosynthesis enzymes (Feinbaum et al., 1991; Batschauer et al., 1996; Noh and Spalding, 1998). Red light (600–700nm) may markedly increase peroxidase (POD) and phenylalanine ammonialyase (PAL) activity in a phytochrome-type response, irritating the aggrandizement of anthocyanin content in the flavonoid metabolic pathways of rice, maize, and turnips (Reddy et al., 1994; Sharma et al., 1999). Some studies have elucidated the effects of different light qualities on anthocyanin accumulation in certain plants and the transcriptome profiling analysis of blueberry fruit has revealed the mechanisms of red and blue light-mediated anthocyanin biosynthesis regulations (Li and Yang, 2007; Lu et al., 2015; Ottosen et al., 2015; Samkumar et al., 2021). However, none of these studies have demonstrated the regulatory mechanism underlying anthocyanin biosynthesis is response to different spectra by integrating the transcriptome and metabolome analysis. This approach may allow us to gain a better understanding of the key genes and metabolites involved in anthocyanin formation under different light qualities in blueberry leaves.

RNA-sequencing is a powerful tool that can be used to unravel novel genes, identify gene expression levels, and facilitate the study of mechanisms underlying metabolite variations (Lv et al., 2019; Zhang Y. et al., 2019). However, it is still difficult to determine a direct correlation between transcript abundance and the associated levels of the respective metabolites since numerous variables are often considered (Lou et al., 2014). Metabolomics is an important part of systematic biology that focuses on the quantitative analysis of all metabolites in an organism to understand the correlations between phenotype and the metabolite (Zhang X. et al., 2019; Guo et al., 2020). An integrative analysis of the transcriptome and metabolome, to investigate the transcript levels in conjunction with the metabolic products, could contribute to the identification of functional genes and the elucidation of pathways involved in plant metabolism processes (Lou et al., 2014).

In this study, an integrated transcriptome and metabolome analysis was used to understand the key genes and metabolites associated with anthocyanin biosynthesis in blueberry leaves under different light qualities. The differential levels of anthocyanin metabolites and their regulatory genes in blueberry leaves were identified under four light qualities, i.e., blue (B), red (R), red/blue (60R/40B), and white light. The connection network was mapped based on a correlation analysis between transcript expression and metabolite levels to highlight the regulator genes and metabolites related to anthocyanin accumulation under different light qualities. Moreover, a correlation analysis between anthocyanin accumulation and photosynthesis was also conducted. The data presented herein will not only provide novel insights into the molecular mechanisms underlying the biosynthesis and regulation of anthocyanins in blueberry, but also useful insights to aid in the breeding of new blueberry varieties with enhanced anthocyanin content.

## Materials and methods

### Plant materials

In this study, three-year-old southern-highbush blueberries of the cultivar ‘Misty’ were utilized, and the plants were grown in a plant factory system with artificial lighting (PFAL) at Zhuang-hang Comprehensive Experimental Station of Shanghai Academy of Agricultural Sciences, Shanghai, China. The growth conditions in the PFAL were as follows: the temperature was set at 25 ± 2°C during the daytime and 22 ± 2°C at night, the photoperiod was 16-h light and 8-h dark, and the CO_2_ concentration and relative humidity were set at approximately 400 μmol·mol^-1^ and 65% ± 5%, respectively. The blueberry plants were treated respectively with four light qualities: white (W), full blue (B), full red (R), and 60% red + 40% blue (60R/40B), and the white light treatment was considered the control. The light intensity of all treatments was equal at 200 ± 5 µmol·m^-2^·s^-1^, each treatment with four experimented blueberry plants. Approximately 30 days after the treatments, the fifth to tenth mature leaves, which were similar developmental stage, had no disease, no mechanical damage, and complete form, were collected from the top branch of each blueberry plant. Approximately 3 g of leaves were collected from each plant and immediately frozen in liquid nitrogen and stored at −80°C for transcriptome profiling and metabolite quantitative analysis. The determination of physiological indicators was performed with four replicates and each plant as a replicate. The transcriptome and metabolome were analyzed with three biological replicates.

### Determination of anthocyanin content

Anthocyanin content in the fresh leaf samples was determined according to the method improved by Li et al. (2016). Leaves were harvested from the blueberry plants, quickly weighed, and homogenized with a mortar and pestle; they were then treated with 0.1 mol·L^−1^ acidized ethanol (10mL) in a microcentrifuge tube and then with water at 60°C for 30 min. The extraction procedure was performed twice and then the samples were centrifuged at 1,500g for 5 min at 4°C, and the supernatants were then collected. The absorbance of the samples was measured at 530, 620, and 650 nm, respectively, using an ultraviolet–visible spectrophotometer (UV-2000) (Spark, TECAN, Switzerland). The values are reported as (1) ΔOD = (OD530 − OD620) − 0.1 (OD650 − OD620) and (2) anthocyanin content = (ΔOD × V × 1000000)/(ξ× m), where V is the dilution volume, ξ is the molar absorption coefficient, and m is the fresh weight of the sample. All measurements were performed with four replicates.

### Leaf photosynthesis measurements

The photosynthesis of the blueberry leaves was measured using a leaf gas exchange system (CIRAS-3; PP Systems). During the measurements, the gas exchange system controlled the environmental conditions within the curette. The temperature of the leaf was 25.0 ± 0.2°C and the CO_2_ concentration was 390 ± 5 μmol·mol^-1^. To discover the photosynthetic efficiency of the different spectra, light response curves were constructed for the blueberry leaves under corresponding light qualities. Photosynthesis indicators, including the maximum gross assimilation rate (*A*_g, max_) and maximum quantum yield for CO_2_ assimilation (*QY*_m, inc_), were calculated according to the light response curves.

### Total RNA extraction, RNA-seq, and transcript profile analysis

Total RNA from the blueberry leaves was isolated using the Polysaccharides and Polyphenolics-rich RNA Prep Pure Plant Kit (TIANGEN, China), following the manufacturer’s instructions. The integrity of the RNA was verified using an Agilent 2100 Bio analyzer (Agilent Technologies, USA). The RNA-Seq and libraries were constructed using the Biomarker Technologies Corporation (Beijing, China) and the Vcorymbosum_v1.0 genome was used as the reference genome in this study. Before conducting data analysis, it is vital to ensure that the red reads are of sufficient quality. Remove reads containing joints and low-quality reads (N was more than 10%; base number with mass value Q≤ 10 accounted for more than 50%), clean reads with high quality were obtained. StringTie was used to assemble the clean reads above and construct a traffic network using the alignment information to construct a multivariable shear map through using the maximum flow algorithm to assemble reads and evaluate its expression. Using the FPKM (Fragments Per Kilobase of transcript per Million fragments mapped) as an indicator to measure the expression level of transcripts or genes through the maximum flow algorithm. The edgeR was used to analysis significant difference of genes transcript level and gene with an adjusted FDR < 0.01& Fold Change ≥2 was identified as differentially expressed. The functions of the unigenes were determined by aligning them to protein databases using BLASTx, including NCBI non-redundant (Nr) and nucleotide (Nt), Swiss-Prot, Kyoto Encyclopedia of Genes and Genomes (KEGG), Clusters of Orthologous Groups of proteins (COGs), and Gene Ontology (GO) databases. GO classification was performed by mapping the relationship between the Swiss-Prot and GO terms, and the genes were mapped to the KEGG database to annotate their potential metabolic pathways (Kanehisa et al., 2008).

### Expression validation of the differentially expressed genes with real-time quantitative RT-PCR

Total RNA was isolated from the blueberry leaves collected under the four light-quality treatments. For the qRT-PCR analysis, the SYBR RNA RT-PCR Kit (TaKaRa, Japan) was used to synthesis the first-strand of cDNA and the Light Cycler 480 (Roche, USA) was used to perform the qRT-PCR procedure. qRT-PCR was performed with the SYBR-Green PCR kit (TaKaRa, Japan) and the reaction mixture system was prepared according to the manufacturer’s instructions. The qPCR program was previously described by Zhang et al. (2021b). The expression level of each gene was calculated in triplicate based on the 2^-ΔΔCt^ algorithm, and *VcGAPDH* was used as the internal reference gene. Snap Gene software (www.snapgene.cn) was used to design the primers for the target genes (**Table S1**).

### Metabolite profiling using ultraperformance liquid chromatography tandem mass spectrometry

Anthocyanins are secondary metabolites in plants. The qualitative and quantitative analysis of the anthocyanins could be measured using the LC-MS/MS system. Freeze-dried blueberry leaves (50 mg) were crushed and dissolved in 500 μL of extract. They were then centrifuged at 1200 g/min for 10min; the extracts were filtrated and absorbed for LC-MS/MS analysis. The instrument system used for data acquisition included an Ultra Performance Liquid Chromatography (UPLC) and Tandem Mass Spectrometry (MS/MS). Qualitative analysis of the anthocyanins detected using mass spectrometry was performed based on the construction of standard databases and the quantification of anthocyanins was identified by employing scheduled multiple reaction monitoring (MRM). The Analyst 1.6.3 software (Sciex) was used to acquire and dispose data and the quantities of all metabolites were analyzed using Multiquanta 3.0.3 software (Sciex). To draw standard curves for the different substances the concentration was used as the abscissa and the peak area as the ordinate. The integrated peak areas for all detected samples were substituted into the standard curve linear equation for calculation and the absolute content values for each sample were obtained.

### Targeted selection of anthocyanins and their intermediates

Targeted anthocyanins and their intermediates were selected based on the following criteria: (1) the obtained compounds should be related to the phenylpropanoid and anthocyanin biosynthesis pathways and their molecular formula and mass information was established using the KEGG and plant metabolic network databases; (2) certain filtrated daughter ions in the anthocyanins could be identified as similar compounds as described in the literary references; and (3) the MS^2^ spectra of the selected metabolites and standard compounds was as found in the METLIN and LIPDMAPS databases. The obtained anthocyanin-related compounds are shown in **Supplementary Table S2**.

### Integrated transcriptome and metabolome analysis

Pearson’s correlation coefficient (PCC), a statistical method used to measure the linear relationship between two variables with values between −1 and 1, was used to calculate the correlation coefficient between the transcriptome and metabolome data and further integration to analyze the relationships between the gene transcript and metabolite content. Associations between PCC > 0.8and *P* < 0.05 were selected and the network of genes and metabolites was constructed using Cytoscape software (Cytoscape Consortium, San Diego, CA). Additionally, genes and metabolites related to anthocyanin biosynthesis were mapped using the KEGG pathway database.

### Data analysis

For this study, all experiments were performed in triplicate and the results are expressed as the mean ± standard deviation (SD). Analysis of variance (ANOVA) was performed to estimate the significant differences between the treatment means at *P* < 0.05.* A* heat map was constructed to exhibit the gene expression pattern and metabolite accumulation and to separate the groups of genes and metabolites with similar expressions and accumulation values. Principal component analysis (PCA) was performed to realize the metabolite variability among the white, blue, red, and red/blue light treatment groups.

## Results

### Phenotypic characteristics, anthocyanin content, and photosynthesis of blueberry leaves under different light qualities

**Figure 1:**
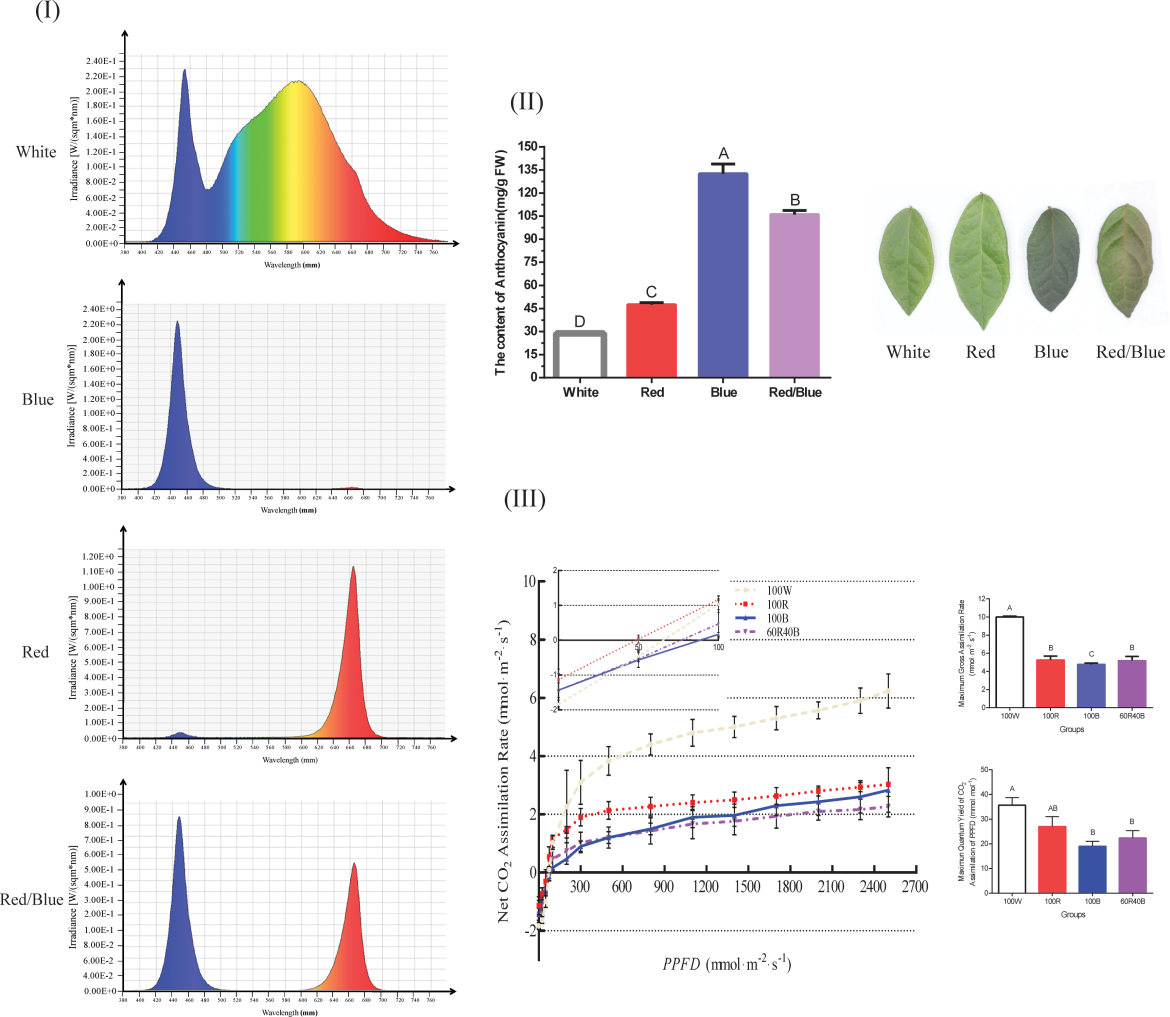
Spectrum plot showing the different light qualities and anthocyanin content, light response curves, and photosynthesis of blueberry leaves under four light spectra. **(I)** Dynamic distributions of the relative spectra under different wavelengths; **(II)** Anthocyanin content and phenotypic comparisons of blueberry leaves under four light spectra; **(III)** Net assimilation (*A*n)-light response curves, *A*_g, max_, and *QY*_m, inc_ for blueberry leaves under different light spectra. *A*_g, max,_ maximum gross assimilation rate; *QY*_m, inc_, maximum quantum yield of CO_2_ assimilation; Capital letters indicate significant differences at *P* < 0.01 among the different treatments. Error bars indicate the standard deviation based on three replicates.

The relative spectra under different wavelengths were significantly different (**Figure 1I**) and the different light qualities influenced the color gradation and anthocyanin synthesis in the blueberry leaves. The color gradation in the blueberry leaves was deepest with the blue light, followed by the red/blue, red, and white lights, and the corresponding anthocyanin content was highest under the blue light, followed by red/blue, red, and white lights. The anthocyanin content of the leaves under blue, red/blue, and red lights was approximately 4.6-, 3.7-, and 1.6-fold higher, respectively, than that under the white light (the control group) (**Figure 1II**). A phenomenon was identified in which the blue and red lights could promote the accumulation of anthocyanin, but differed significantly in their impact levels. The promotion of anthocyanin biosynthesis was more prominent under blue light than red light, while the accumulation was in between these two levels with the red/blue light combination. Leaf photosynthesis exhibited adverse effects on anthocyanin accumulation, which were strongest under white light but lowest under blue light. The photosynthesis indices, including *A*_g, max_ and *QY*_m, inc_, presented a consistent trend, with the greatest index under the white light, followed by red, red/blue, and blue (**Figure 1III**). Blue and red lights can promote anthocyanin accumulation but impair photosynthesis in blueberry leaves. In contrast, white light prejudiced anthocyanin biosynthesis but facilitated photosynthesis.

### Transcriptome data and screenings for the DEGs under different light qualities

To elucidate the molecular genetics mechanisms underlying anthocyanin biosynthesis in blueberry leaves when under different light conditions, a comparative transcriptome analysis was performed. The Illumina sequencing platform was used to construct and sequence twelve RNA-Seq libraries (each treatment with three biological replicates). The rigorous quality estimation and data cleaning resulted in 21.48–26.23 M clean reads with Q30 bases that were identified as high-quality reads for further analysis. Their GC contents and Q30 ranges were 46.93%–47.36% and 94.69%–94.93%, respectively (**Table S3**). Moreover, the N percentage was 0.00% in all samples.

**Figure 2:**
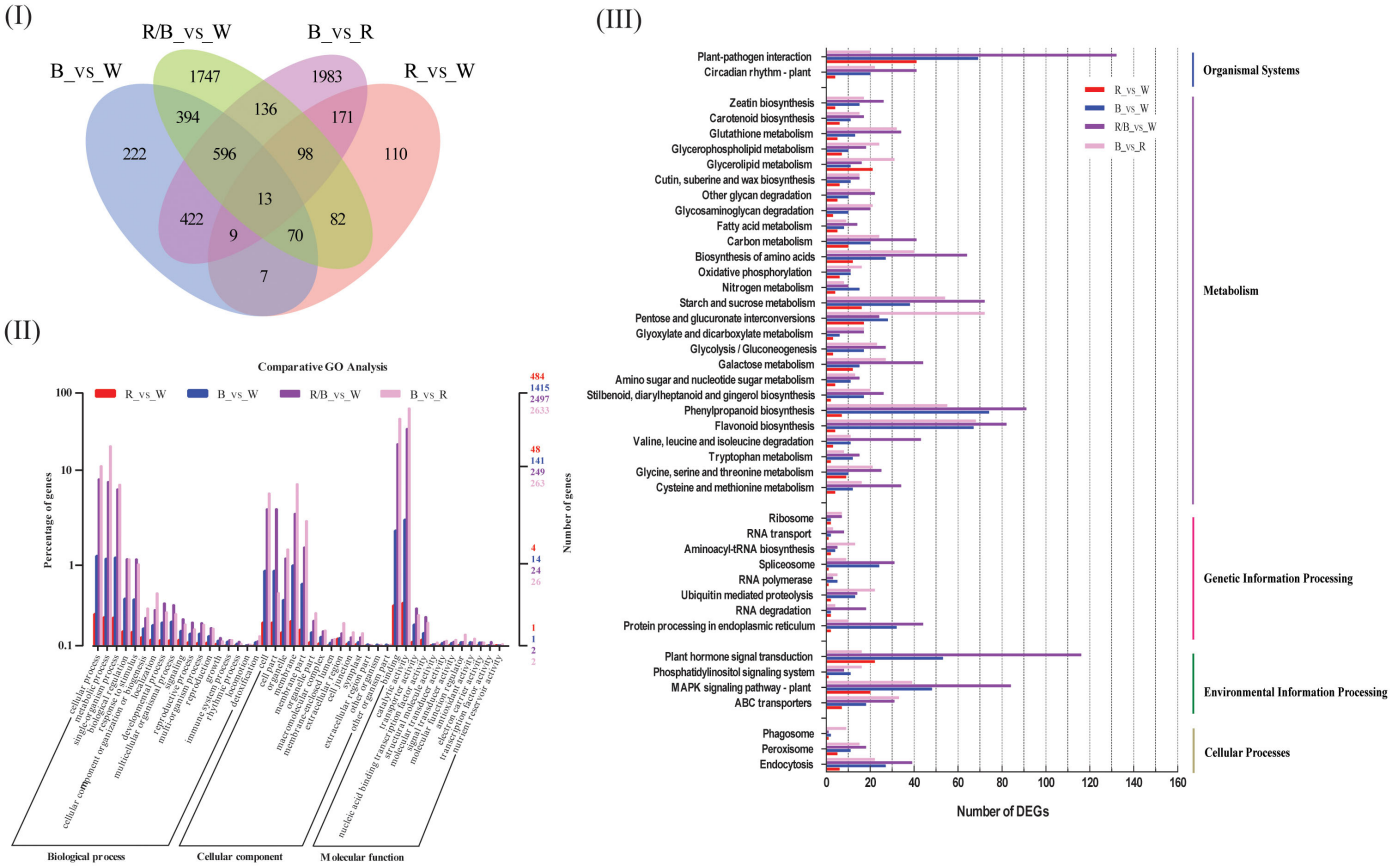
Functional annotation results of the genes differentially expressed in blue **(B)**, red **(R)**, and red/blue (60R/40B) when compared with those of the corresponding control (white light) and the comparison of B vs. R using the transcriptome data from blueberry ‘Misty’ leaves; **(I)** Venn diagram showing the shared and common DEGs for all light quality treatments; **(II)** Functional annotation of the DEGs based on the Clusters of Orthologous Groups (COG) database; **(III)** Annotation information obtained from the Gene Ontology (GO) database; **(IV)** Functional classification of DEGs based on the Kyoto Encyclopedia of Genes and Genomes (KEGG) dataset.

To substantiate the effects of light quality on the transcript levels of anthocyanin biosynthesis genes in blueberry leaves, DEGs between samples from the light quality treatments were identified using pairwise comparisons of the four cDNA libraries (blue vs. white, red vs. white, red/blue vs. white, and blue vs. red), and the fragments per kilo-base per million mapped reads (FPKMs) of all DEGs were calculated. A total of 1812 (640 up-regulated and 1172 down-regulated), 596 (250 up-regulated and 346 down-regulated), 3283 (1123 up-regulated and 2160 down-regulated), and 3428 (1974 up-regulated and 1457 down-regulated) differentially expressed genes (P < 0.05, fold change > 2) were found during the comparisons of the blue and white, red and white, red/blue and white, and blue and red libraries, respectively, whereas 13 DEGs were shared among the four comparisons (**Figure 2I**; **Table S4**). The number of DEGs was substantially higher in the blue vs. white comparison when compared with red vs. white, which indicated a strong gene expression disturbance experienced by the blueberry leaves under blue light. The blueberry leaves were more sensitive and actively responded to blue light to promote anthocyanin accumulation when compared to red light. GO term annotation was performed to improve our understanding of the functions of these DEGs in blueberry leaves under different light conditions. The DEGs were considered enriched in GO-terms if at least one of the terms was categorized as a biological process, cellular component, or molecular function (**Figure 2II**). The results of the GO-biological process (GO-BP) analysis revealed that significantly enriched DEGs were associated with metabolic processes, mainly phenylalanine and secondary metabolisms, among the four comparisons. To further explore the enriched metabolic pathways related to anthocyanin accumulation in blueberry leaves, we mapped all DEGs to reference canonical pathways in the KEGG database. A total of 4097 DEGs (blue vs. white:844; red vs. white:307; red/blue vs. white:1440; and blue vs. red:1506) with annotated KEGG results were classified into five main categories, including organismal systems, metabolism, genetic information processing, environmental information processing, and cellular processes, based on the type of KEGG pathways (**Figure 2III**). During metabolism classification, phenylpropanoid biosynthesis and starch and sucrose metabolism (photosynthesis) were the primary enriched pathways throughout the four comparisons. Additionally, DEGs were annotated by searching against the COG dataset. COG-annotated putative proteins were functionally classified into at least 26 categories, including cellular structure, biochemical metabolism, molecular processing, and signal transduction (**Figure S1**). In addition, the DEGs were collected and classified into 17 groups based on their annotated functions (**Table 1**). Among all the categories, phenylalanine metabolism, secondary metabolism, starch and sucrose metabolism, and carbohydrate metabolism (photosynthesis) were highly significant and enriched pathways, as well as being enriched they also had a greater number of DEGs. The results indicated that these DEGs were associated with metabolic pathways that could contribute to the biosynthesis and accumulation of anthocyanin in blueberry leaves under different light qualities.

**Table 1:** Functional categories of the genes differentially expressed in the blue, red, and red/blue light when compared with white light and the comparison of blue vs. red in blueberry leaf.

Annotation	Blue_vs_White				Red_vs_White				Red/Blue_vs_White				Blue_vs_Red			
	Up		Down		Up		Down		Up		Down		Up		Down	
	Number	%	Number	%	Number	%	Number	%	Number	%	Number	%	Number	%	Number	%
Photosynthesis	5	0.74	1	0.08	2	0.73	1	0.29	5	0.40	2	0.09	3	0.22	8	0.39
Cell part	160	23.53	290	23.09	68	24.82	72	20.69	307	24.74	516	22.64	340	25.70	577	27.79
Secondary metabolism	23	3.38	103	8.20	22	8.03	21	6.03	37	2.98	167	7.33	67	5.06	117	5.64
Plant hormone	17	2.50	36	2.87	12	4.38	10	2.87	45	3.63	71	3.12	7	0.53	9	0.43
Signaling	20	2.94	39	3.11	3	1.09	18	5.17	28	2.26	65	2.85	26	1.97	13	0.63
Cell	160	23.53	290	23.09	68	24.82	72	20.69	307	24.74	516	22.64	340	25.70	577	27.80
Development	40	5.88	85	6.77	10	3.65	21	6.03	80	6.45	148	6.49	83	6.27	121	5.83
Growth	3	0.44	16	1.27	2	0.73	8	2.30	12	0.97	23	1.01	7	0.53	23	1.11
Carbohydrate metabolism	43	6.32	58	4.62	31	11.31	11	3.16	89	7.17	118	5.18	65	4.91	134	6.45
Transport activity	43	6.32	85	6.77	5	1.82	19	5.46	52	4.19	174	7.63	71	5.37	116	5.59
Starch and sucrose metabolism	19	2.79	19	1.51	4	1.46	12	3.45	31	2.50	41	1.80	20	1.51	34	1.64
Phenylalanine metabolism	5	0.74	12	0.96	1	0.36	0	0.00	2	0.16	16	0.70	7	0.53	4	0.19
Biological regulation	105	15.44	199	15.84	32	11.68	70	20.11	189	15.23	380	16.67	232	17.54	289	13.92
Molecular function regulator	21	3.09	17	1.35	7	2.55	11	3.16	26	2.10	25	1.10	37	2.80	32	1.54
Function unknown	8	1.18	4	0.32	5	1.82	0	0.00	15	1.21	4	0.18	10	0.76	9	0.43
RNA regulation	8	1.18	2	0.16	2	0.73	2	0.57	16	1.29	13	0.57	8	0.60	13	0.63
Total	680	100.0	1256	100.0	274	100.0	348	100.0	1241	100.0	2279	100.0	1323	100.0	2076	100.0

### Transcriptional profiles of the DEGs related to photoreceptors, photosynthesis, and anthocyanin metabolism in blueberry leaves under different light qualities

**Figure 3:**
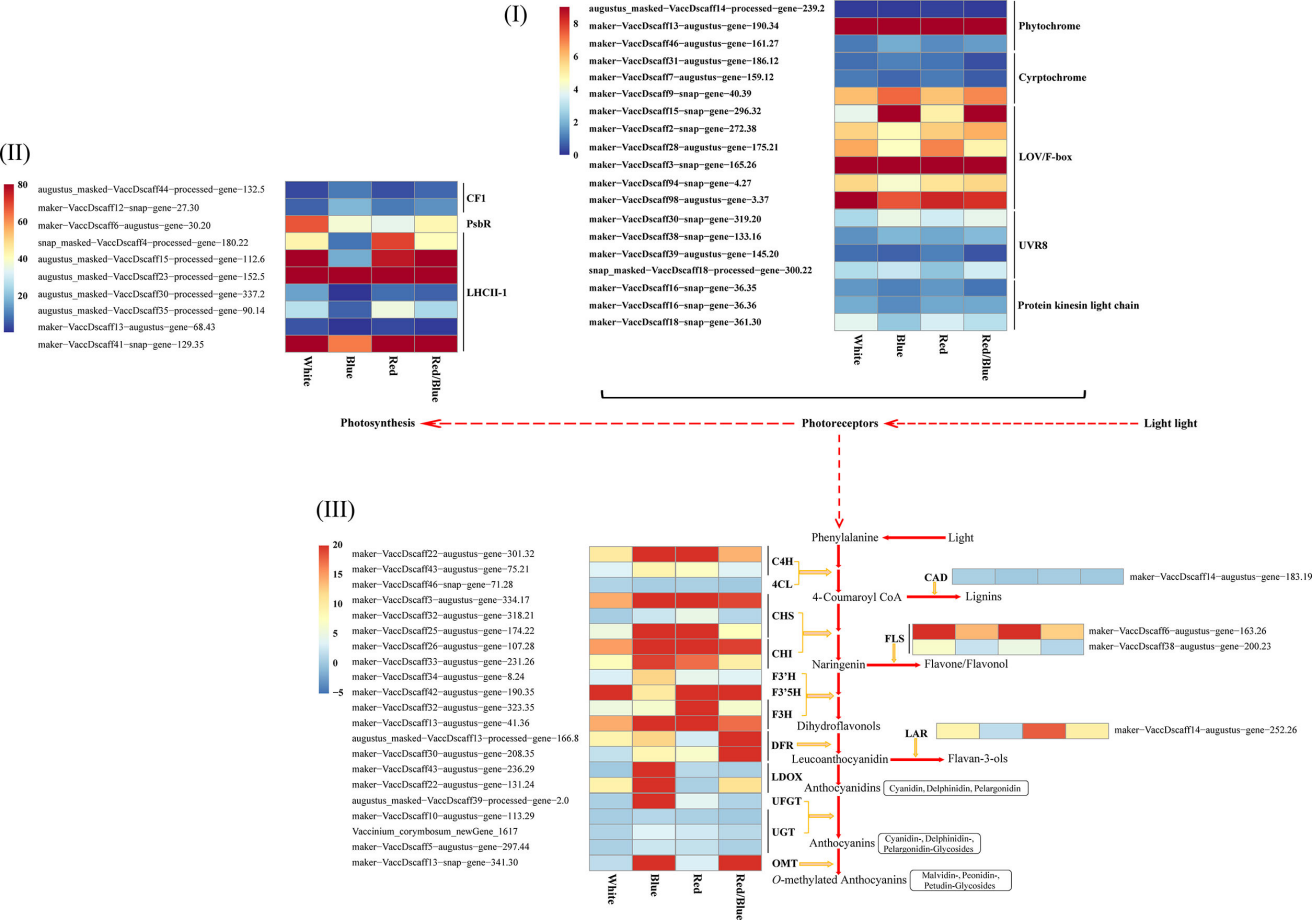
Expression heatmap showing the differentially expressed genes related to photoreceptor, photosynthesis, and anthocyanin and flavonoid biosynthesis pathway under different light qualities for the blueberry leaf. **(I)** Differential expression of the genes related to the photoreceptor, including phytochrome, cryptochrome, LOV/F-box, UVR8, and protein kinases in the light chain; **(II)** Expression levels of the DEGs from CF1, PsBR, and LHCII-1; **(III)** Expression patterns of the DEGs involved in the anthocyanin and flavonoid biosynthesis pathway. Enzyme names and expression levels are shown at the side of each step. C4H, cinnamate 4-hydroxylase; 4CL, 4-coumarate:CoA ligase; CHS, chalcone synthase; CHI, chalcone isomerase; OMT, *O*-methyltransferase; F3′H, flavonoid3′-monooxygenase; F3′5H, flavonoid 3′, 5′-hydroxylase; DFR, dihydroflavonol-4-reductase; LDOX, leucoanthocyanidin dioxygenase; LAR, leucoanthocyanidin reductase; UFGT, UDP-glucose flavonoid glucosyltransferase; UGT, anthocyanidin 3-O-glucoside 5-O-glucosyltransferase; CAD, cinnamyl alcohol dehydrogenase; FLS, flavonolsynthase; Red and blue, respectively, indicated high and low expression according to the color bar using the unit variance scaling method.

The transcriptome analysis under light conditions showed that the DEGs related to phenylalanine, secondary, starch and sucrose, and carbohydrate (photosynthesis) metabolism processes may help blueberry leaves to accumulate anthocyanins. The physiological indicators suggested that there were significant differences in anthocyanin accumulation and photosynthesis in blueberry leaves due to the distinct light qualities. The DEGs implicated in the crucial steps of these processes were consequently screened. Moreover, the blueberry leaf was treated with distinct spectra, the DEGs associated with photoreceptor responses to the different light qualities were searched for. Core genes related to anthocyanin biosynthesis, photosynthesis, and photoreceptors were studied in detail, and the results demonstrated that most of the uni-transcripts showed significant changes in their expression levels (**Figure 3**; **Table S5**). An opposite dynamic trend was observed between the expression patterns of the anthocyanin accumulation-related and photosynthesis-related DEGs. The expression levels for most of the anthocyanin biosynthesis-related DEGs were higher in the blue light when compared with the other lights, but the DEGs associated with photosynthesis were down-regulated with the blue light. There may be feedback regulation among the DEGs related to anthocyanin accumulation and photosynthesis under blue light. DEGs encoding phytochrome, as receptor proteins of red and far-red light (600–750 nm), were up-regulated under red or red/blue light. Consistently, DEGs encoding cryptochrome, which mediated several responses to blue light and UV-A (320–500 nm), were up-regulated under blue light (**Figure 3I**). In relation to photosynthesis, there were DEGs among the different spectra. Photosynthesis-related DEGs were down-regulated under blue light when compared with the other treatments (**Figure 3II**), which was consistent with the weakness of photosynthesis under blue light. These results suggest that blue light prejudiced photosynthesis in the blueberry leaves, which is in sharp contrast to the results for anthocyanin accumulation under blue light. In many cases, changes in anthocyanin biosynthesis correspond to changes in the expression of genes that encode pathway enzymes. Among the related anthocyanin accumulation processes, 22 DEGs (including *C4H*, *4CL*, *CHS*, *DFR*, *F3’H*, *F3’5H*, *F3H*, *LDOX*, *OMT*, and *UFGT*) were found to participate in vital steps in the anthocyanin pathway. Among these, *LDOX*, *UFGT*, and *OMT* were expressed well under blue light, whereas *DFR* and *OMT* were highly expressed under red/blue light. These DEGs contributed to the cyanidin, pelargonidin, malvidin, delphinidin, petunidin, and peonidin accumulation in the anthocyanin biosynthesis pathway (**Figure 3III**). Additionally, the expression of the DEGs related to lignin and flavonoid metabolism, which are also significant secondary metabolite pathways in plants, decreased in the blue light in comparison with that in white, red, and red/blue lights. These results indicate that blue light could promote the accumulation of anthocyanin but hinder the photosynthesis and secondary metabolism process of lignin and flavonoids in blueberry leaves when compared with other light sources *via* the direct or indirect regulation of the expression levels of related DEGs.

### Differentially expressed transcription factors and the co-expression network between DETFs and DEGs involved in anthocyanin and flavonoid accumulation

**Figure 4:**
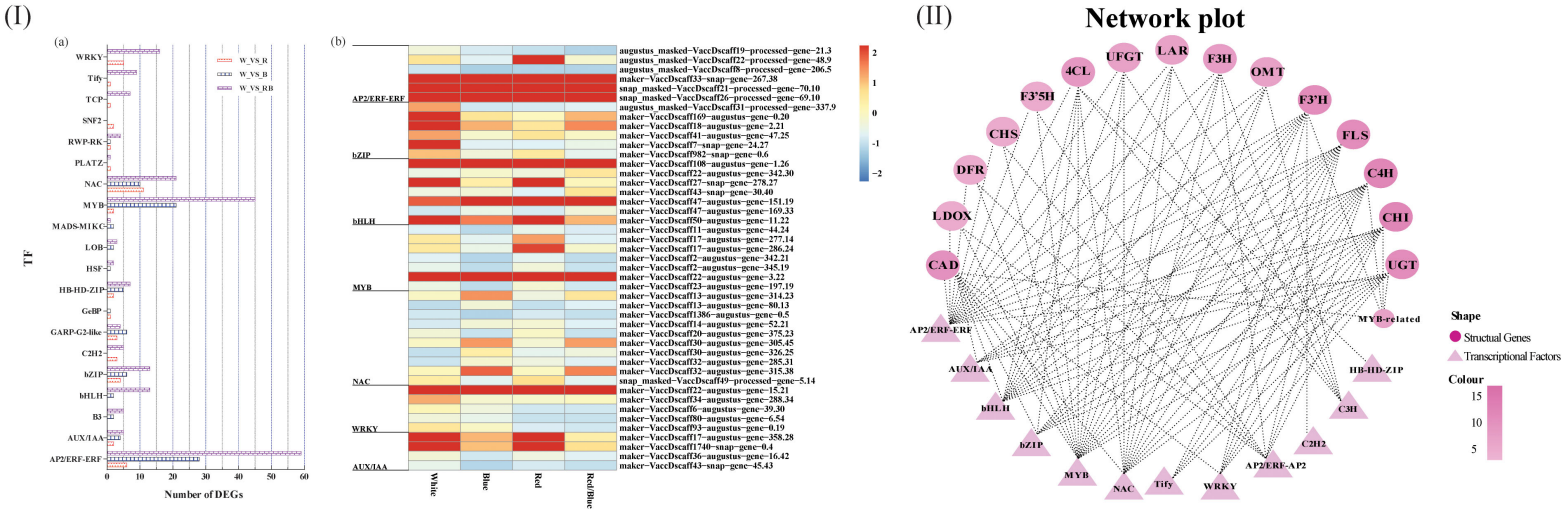
Distribution of the differentially expressed transcription factors (TFs) and co-expression networks between candidate genes and transcription factors. **(I)** Quantitative statistics for the differentially expressed TFs **(A)** and a heatmap showing the candidate TFs under various light qualities **(B)**; **(II)** Co-expression networks between differentially expressed structural genes involved in the anthocyanin and flavonoid biosynthetic pathway and differentially expressed TFs under different light spectra.

Blueberry leaves under different light qualities showed significantly different TFs, belonging primarily to 20 different families (**Figure 4I**; **Table S6**). The differentially expressed TFs belonged mostly to the AP2/ERF-ERF, bZIP, bHLH, MYB, and NAC families. The number distribution mole for the TFs in the blueberry leaves under different light qualities was consistent with the quantitative distribution of the gene function annotation. There were more differentially expressed genes or TFs under the red/blue light, indicating that the red/blue light combination was beneficial for changes in gene or TF expression levels.

TFs regulate gene expression patterns by acting as activators or repressors to induce or inhibit gene promoter activity. There is a potential regulatory mechanism by which differentially expressed TFs (DETFs) may influence anthocyanin biosynthesis by adjusting the expression levels of the DEGs implicated in anthocyanin accumulation under different light qualities. To verify this hypothesis, co-expression networks were established between the TFs and genes using gene expression profile data based on the correlation coefficient. The resulting co-expression networks indicated that different TFs connected distinct genes, and there were correlations among them (**Figure 4II**). TFs (*MYB*, *NAC*, and *bHLH*) connected more genes related to anthocyanin accumulation, and these TFs were highly expressed in blueberry leaves under blue light.

### Validation of key DEGs and DETFs involved in anthocyanin accumulation by qRT-PCR

**Figure 5:**
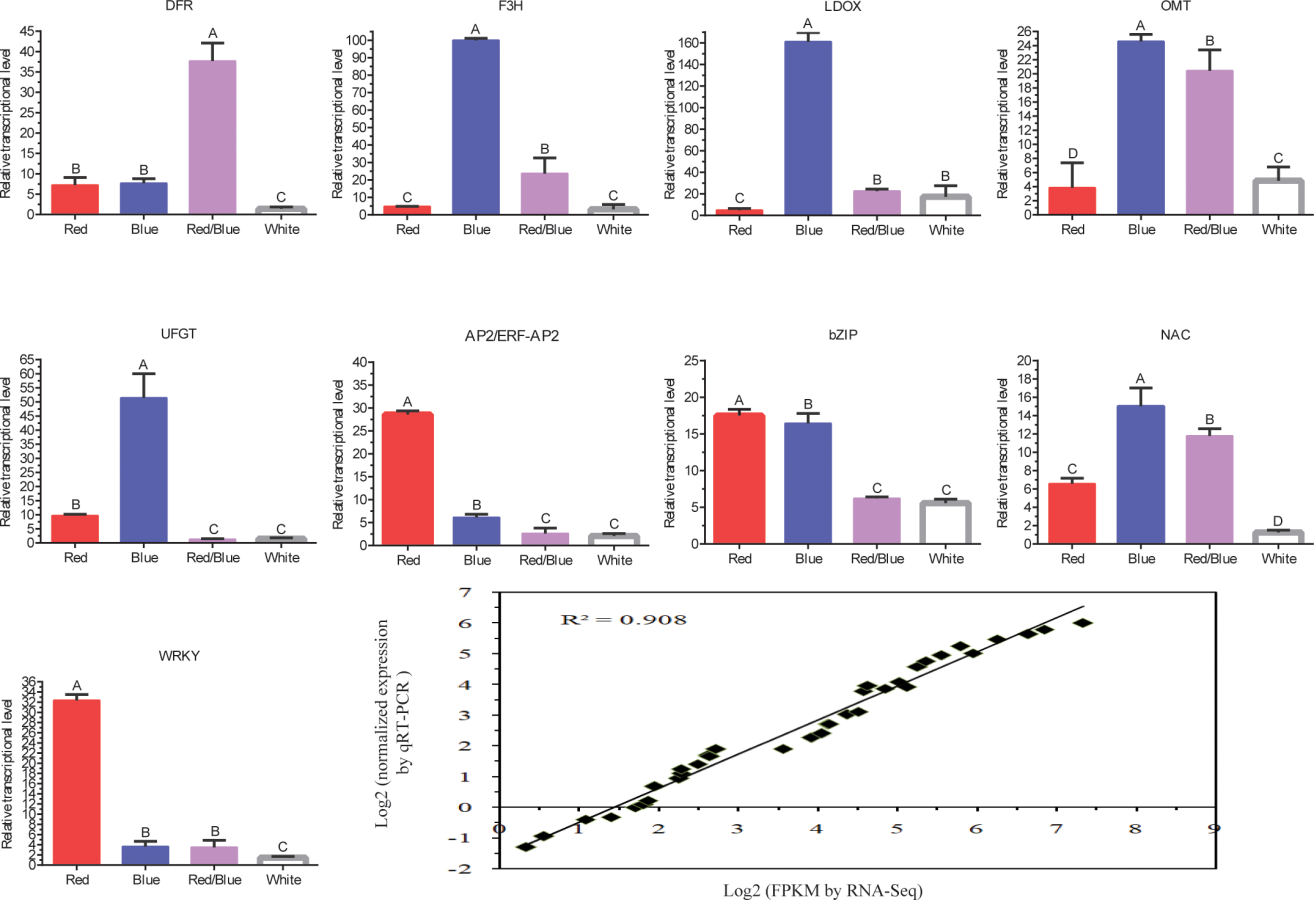
Quantitative RT-PCR validation of the structural genes involved in the anthocyanin biosynthesis pathway and transcription factors and the correlation analysis of the gene expression results obtained from the qRT-PCR and RNA-Seq under different light qualities (red, blue, red/blue, and white). The x-axis represents the different light qualities while the y-axis represents the relative expression of each gene. Capital letters indicate significant differences at *P* < 0.01 among the different treatments. Error bars represent the standard deviation based on three replicates.

To further validate the comparative transcriptome results, the transcript level variances of some putative genes or TFs related to anthocyanin accumulation were evaluated by qRT-PCR analysis with three biological replicates (**Figure 5**). Structural genes, including *F3H*, *LDOX*, *OMT*, and *UFGT*, involved in the anthocyanin biosynthesis pathway exhibited similar dynamic trends, with the highest transcript levels under blue light, followed by red/blue, red, and white. The expression level of *DFR* was the highest under red/blue light, and the effect of inducing *DFR* transcripts was the least significant under white light. The transcript level for *UFGT* was the lowest under red/blue light, and its peak occurred under blue light. Moreover, the transcript level for the *OMT* was second highest under the red/blue light. These expression trends agreed with the alterations in gene expression detected by the transcriptome analysis, corroborating the RNA-sequencing data. These results coincided with the dynamic changes in anthocyanin content under different light qualities (they were highest in blue light, followed by red/blue, red, and white light), indicating that these structural genes may play a role in anthocyanin biosynthesis in blueberry leaves in response to the different spectra. The expression levels of the TFs (*AP2/ERF-AP2*, *bZIP*, *NAC*, and *WRKY*) were higher in the blue, red, and red/blue lights than in the corresponding control (white light). Moreover, a correlation analysis of the gene expression results was obtained using qRT-PCR and RNA-Seq data under different light qualities and a close connection was identified between them (correlation coefficient: R^2 =^ 0.908). The high consistency between the RNA-Seq and qRT-PCR results suggested that the RNA-Seq data were reliable for evaluating the regulation of gene expression under different light quality treatments in blueberry leaves.

### Metabolome profiling analysis of blueberry leaves under different light qualities

**Figure 6:**
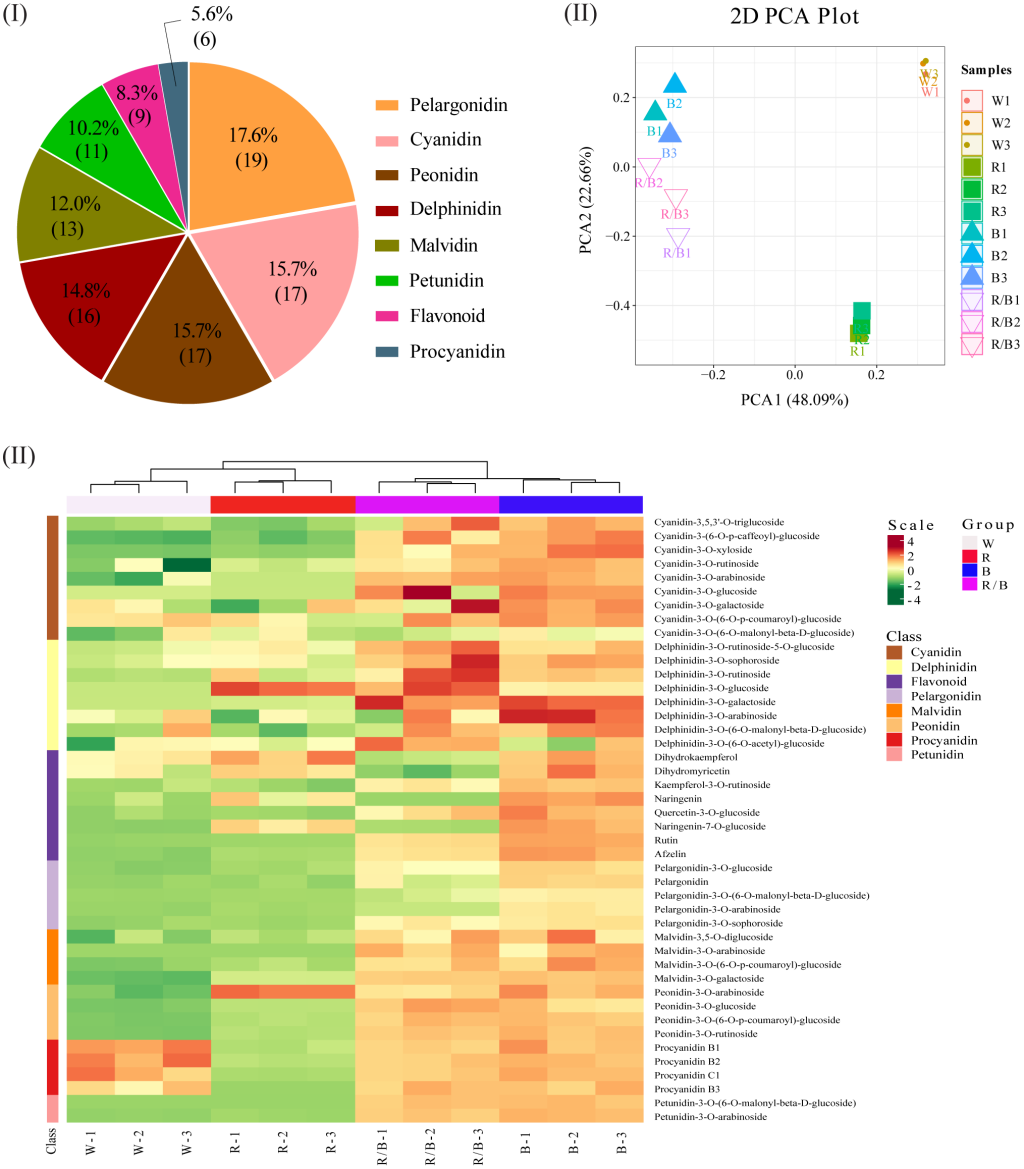
Metabolome profiling of ‘Misty’ blueberry leaves under different light qualities: white (W), red (R), blue (B), and red/blue (R/B). **(I)** Primary classification of the metabolites identified in the leaves. **(II)** Principal component analysis (PCA) scores for the leaves under different light qualities for the first component (PC1) and second component (PC2). **(III)** Hierarchical clustering and correlation analysis of the leaf metabolites under different light qualities.

To compare the metabolite compositions associated with anthocyanin accumulations in the blueberry leaves under different light conditions, the major anthocyanins were determined using LC-MS/MS. In total, 108 anthocyanidins were detected and grouped into eight categories (**Figure 6I**). The largest category (pelargonidin) contained 19 metabolites, followed by cyanidin (n=17), peonidin (n=17), delphinidin (n=16), and malvidin (n=13). The PCA for the anthocyanin derivatives was used to classify twelve samples into four distinct clusters, accurately reflecting the four light quality treatments for the blueberry leaves (**Figure 6II**). In this model, the secondary principal component (PC2; 22.66% of the total variables) was clearly separated between blue and red/blue light. The differences between the white and red light resulted from PC1 and PC2 in this model (48.09% and 22.66% of the variables, respectively). An Orthogonal Partial Least Squares-Discriminant Analysis (OPLS-DA) model was performed to identify the differentially accumulated metabolites responsible for metabolic differentiation among the various light quality treatments for the blueberry leaves. A total of 43 (25 up-accumulated; 3 down-accumulated), 41 (7 up-accumulated; 4 down-accumulated), 44 (24 up-accumulated; 3 down-accumulated), and 42 (22 up-accumulated; 1 down-accumulated) metabolites were selected for comparison analysis as follows: blue vs. white, red vs. white, red/blue vs. white, and blue vs. red lights, respectively (**Table S7**). The hierarchical clustering and correlation analysis exhibited a compact dependence of the anthocyanin metabolites on the different light qualities of the blueberry leaves (**Figure 6III**). The overall accumulation of the anthocyanin metabolites was higher under blue light than under the other light conditions, and the accumulation of cyanidin, pelargonidin, malvidin, peonidin, and petunidin was significantly higher under the blue and red/blue lights when compared with the white and red lights.

### Differential accumulation of derivatives related to anthocyanin biosynthesis under different light qualities

**Figure 7:**
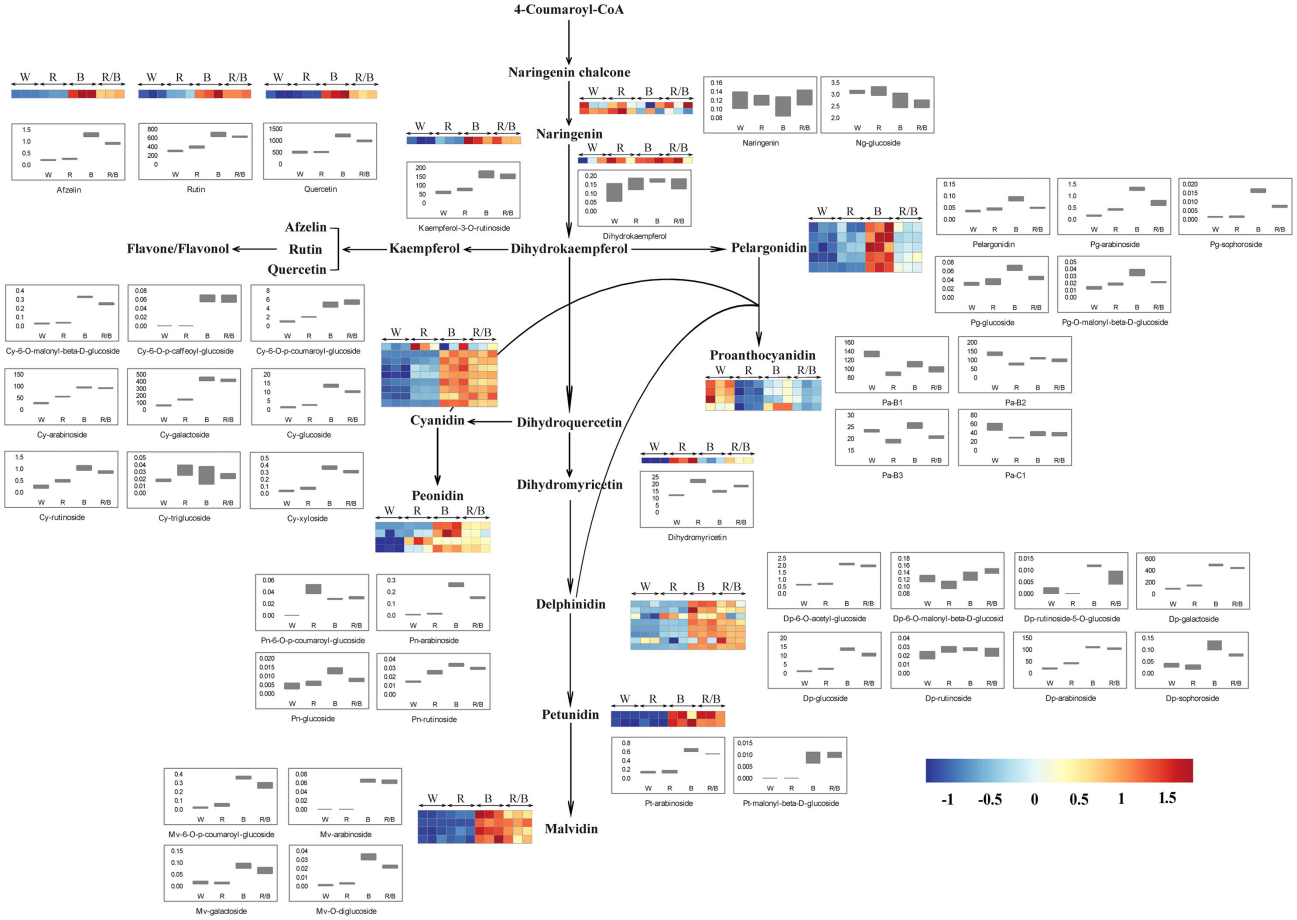
Model showing the changes in the abundance of anthocyanin and flavonoid biosynthesis pathway metabolites in blueberry leaves under different light qualities. The model was constructed based on the KEGG pathway and a literary reference. Ng, naringenin; Pg, pelargonidin; Cy, cyanidin; Pa, proanthocyanidin; Pn, peonidin; Dp, delphinidin; Pt, petunidin; Mv, malvidin; W, white; R, red; B, blue; R/B, Red/Blue; Each colored unit represents the normalized intensity of each compound ion according to the color scale (three biological replicates × four treatments, *n*=12). Box-and-whisker plots show the changes in the metabolites for each treatment. Maximum and minimum values of a metabolite among three biological replicates are represented at the upper and lower ends of the whisker, respectively.

Based on the KEGG database annotation, 44 metabolites were found to be involved in the anthocyanin biosynthesis pathway and the identified anthocyanin derivatives and their relevant compounds were rearranged based on their corresponding positions in the anthocyanin biosynthesis pathway, which were established based on the KEGG, PMN, and literature references. Among them, two naringenins (Ng), one dihydrokaempferol (Dk), one kaempferol (Ka), three flavones, nine cyanidins (Cy), four peonidins (Pn), five pelargonidins (Pg), one dihydromyricetin, eight delphinidins (Dp), two petunidins (Pt), four malvidins (Mv), and four proanthocyanidins (Pa) were identified (**Figure 7**). Each metabolite in the anthocyanin biosynthesis pathway differed significantly under the different light conditions in the blueberry leaves. Interestingly, the content for most metabolites was highest under the blue light but lowest under the white light, suggesting that the blue light was beneficial in promoting anthocyanin accumulation in blueberry leaves. The dynamic changes in the content of metabolites in the anthocyanin biosynthesis pathway were consistent with the variations in the expression levels for the genes related to anthocyanin accumulation under the different light qualities. Among all anthocyanin derivatives, the quantification of Cy-type, Pg-type, and Mv-type derivatives were more advantageous under blue light than under other light qualities. However, the combination of blue and red light could dramatically improve the Dp-type, Pn-type, and Pt-type derivative content.

### Integrated analysis of differentially accumulated metabolites and DEGs in response to different light qualities

**Figure 8:**
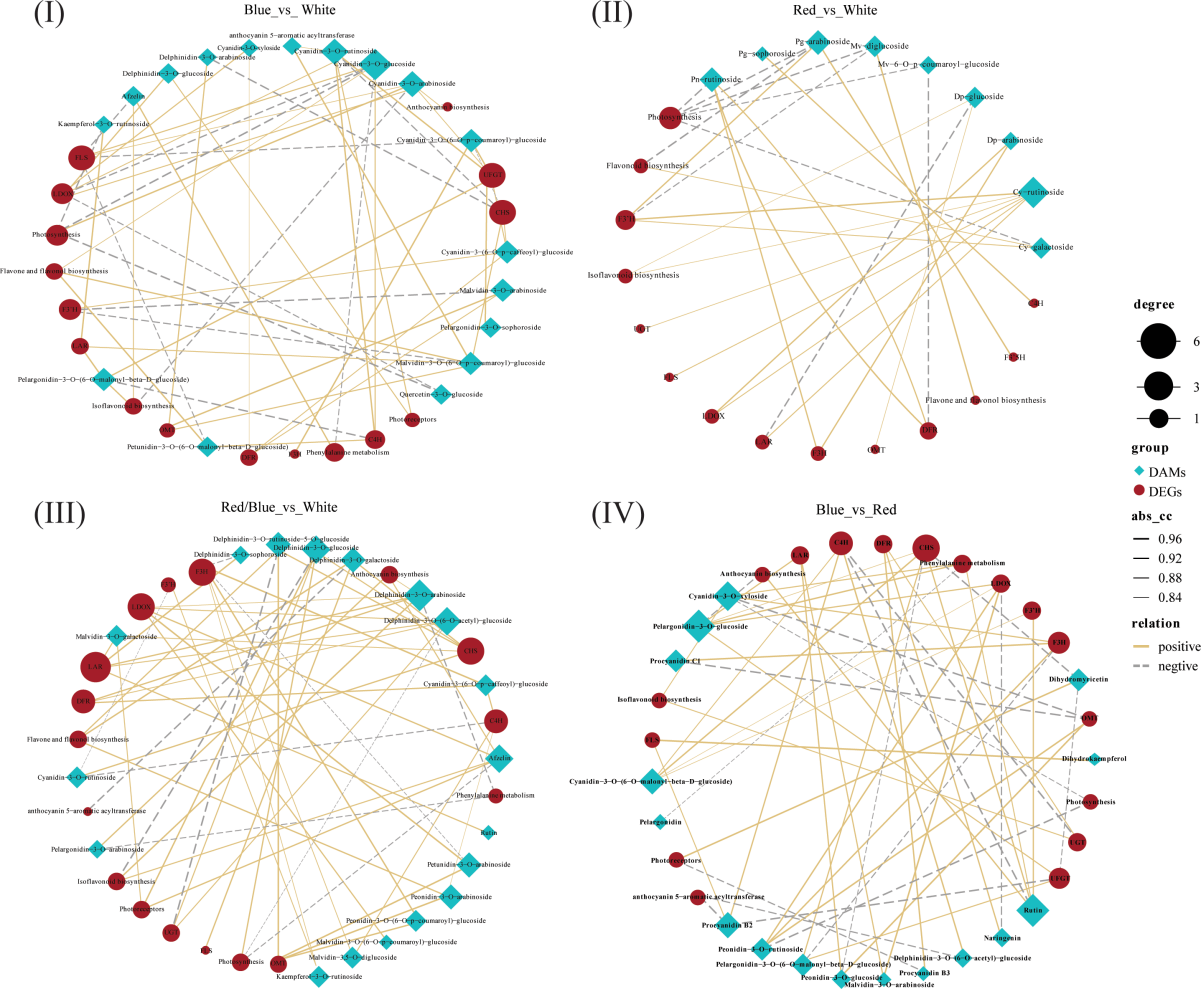
Results of the correlation analysis for the differentially expressed genes (DEGs) and differentially accumulated metabolites (DAMs) involved in the anthocyanin biosynthesis pathway of blueberry leaves based on the correlation coefficient (CC) and the p-value for the correlation coefficient (CCP) between DAMs and DEGs. Analysis of **(I)** blue vs. white, **(II)** red vs. white, **(III)** red/blue vs. white, and **(IV)** blue vs. red. The size of the black dots indicates the number of connected DAMs or DEGs; the width of the connecting lines indicates the strength of the correlation; solid lines indicate positive correlations and dashed lines indicate negative correlations.

To understand the regulatory networks for the anthocyanins that were implicated in the differential distribution of anthocyanin derivatives under the different light qualities, we performed an integrated analysis of the core differentially accumulated metabolites (DAMs) and DEGs related to anthocyanin accumulation in the four comparisons (blue vs. white, red vs. white, red/blue vs. white, and blue vs. red). Based on the above results, the DEGs related to secondary metabolism, carbohydrate metabolism processes(photosynthesis), anthocyanin biosynthesis, and anthocyanin derivatives of Cy, Dp, Ng, Pg, Pa, Pn, Pt, and Mv were used to perform an interaction network based on the Pearson correlation analysis. In this interaction network, genes and metabolites were closely related, and positive and negative correlations between gene expression levels and anthocyanin accumulation content were discovered (**Figure 8**; **Table S8**). In contrast to the comparison between red and white (28), there were much stronger correlations between DAMs and DEGs in both comparisons with blue vs. white (57) and red/blue vs. white (56). This result demonstrated that the blue light or blue/red combination may play a more prominent role in promoting the biosynthesis and accumulation of anthocyanins because there were more DAMs and DEGs related to anthocyanin formation. Because of the changes in DAM content and the DEG transcriptional level, the color and anthocyanin content of the blueberry leaves differed significantly under different light qualities. In the blue vs. white correlation network, there was a close correlation between Cy and *LDOX*, Mv and *OMT*, and Pg and *UFGT* levels. *LDOX*, *OMT*, and *UFGT* are upstream regulatory genes for Cy, Mv, and Pg synthesis in the anthocyanin pathway, and their high expression contributes to Cy, Mv, and Pg accumulation, respectively. Based on the results of the analysis of the DEG transcriptional profile and DAM metabolic profile, the expression levels of *LDOX*, *OMT*, and *UFGT* and the contents of the Cy, Mv, and Pg derivatives were highest under blue light. This validated the idea that blue light induced substantial accumulation of the Cy, Mv, and Pg derivatives *via* the promotion of *LDOX*, *OMT*, and *UFGT* expression, respectively, further facilitating anthocyanin synthesis. A similar phenomenon was also found in the red/blue vs. white correlation network: there were higher expression levels of *DFR* and *OMT*, greater accumulation of Dp, Pn, and Pt derivatives, and an intimate connection between *DFR* and Dp, *OMT* and Pn, and *OMT* and Pt under a combination of red and blue lights. *DFR* and *OMT* induced the synthesis of Dp, Pn, and Pt derivatives, leading to an increase in anthocyanin content under the red/blue light in the blueberry leaves. Additionally, in the blue vs. red correlation network, *UFGT* and *OMT* were closely connected with Pg and Pn, respectively. Among the DEG transcriptional profiles and DAM metabolic profiles with the comparison of blue vs. red, the transcript levels of *UFGT* and *OMT* and the accumulations of Pg and Pn were higher in blue when compared with red. This further indicates that *UFGT* and *OMT* play vital roles in promoting Pg and Pn accumulation, respectively.

## Discussion

Blueberry is a commercially popular small fruit crop with leaves and fruit that are abundant in anthocyanins, which are secondary metabolites considered beneficial for human health (Hou et al., 2011; Norberto et al., 2013; Zhou et al., 2013). Under natural growth conditions, the biosynthesis of anthocyanins is dramatically affected by light, including both light quality and intensity. This occurs *via* regulatory gene activity and key metabolites level associated with anthocyanin biosynthesis that can modulate anthocyanin content and pigmentation activity (Dixon and Paiva, 1995; Chalker-Scott, 1999; Jaakola, 2013; Ottosen et al., 2015; Ma et al., 2019), among which, the light quality plays a more critical role (Briggs and Olney, 2001; Zoratti et al., 2014). However, little is known about the molecular mechanisms underlying the changes in anthocyanin content in response to different light qualities in blueberry leaves. In this study, an integrated analysis of the transcriptome and metabolome during anthocyanin biosynthesis under different light qualities was carried out, which allowed us to gain insight into the key metabolites, genes, and metabolic pathways involved in anthocyanin biosynthesis and accumulation in blueberry.

During the development of green tissues (such as leaves, flower buds, and stems) and tissue-cultured cells, light promotes the accumulation of anthocyanins by activating gene transcript levels related to anthocyanin metabolic pathways (Uleberg et al., 2012; Zhang et al., 2021a). In the early stages of anthocyanin synthesis, the expression patterns of related genes can be regulated by different light qualities and intensities, and this expression pattern is dramatically changed and accompanied by distinct species. For example, anthocyanin accumulation within the blueberry peel is significantly affected by light, the content of anthocyanin and expression level of genes related to anthocyanin synthesis pathway were significantly different under various light treatments including: intensity 100%, 50%, and 20% (Guo et al., 2021). Moreover, the differences in the light qualities on the anthocyanin biosynthesis also were significant. Compared with the white control film, the red and yellow films led to a substantial increase in the total anthocyanin content (TAC), while the green and blue films caused a decrease in TAC. The colored film treatment also significantly affects the related enzyme activity and the expression of structural genes and transcription factors in strawberry fruits (Miao et al., 2016). Other studies have shown that the anthocyanin content of purple celery was significantly higher with the blue light treatment when compared with the other light quality conditions (red, green, yellow, and fluorescent lamps) (Field et al., 2001; Neill and Gould, 2003; Hughes et al., 2005). In this study, we measured the anthocyanin content in blueberry leaves under different light qualities and found that the maximal accumulations occurred with blue light, followed by red/blue, red, and white (control). Compared with the white light, the blue, red, and red/blue combination could markedly enhance the accumulation of anthocyanin, but the effects of the other light qualities varied. Blue light was more favorable for the accumulation of anthocyanin than red, red/blue, and white light in blueberry leaves, which is consistent with a previous report on wild-type petunias, in which blue light radiation significantly increased the anthocyanin content in leaves (Nick et al., 2009; Tao et al., 2018), while red light has been reported to promote anthocyanin accumulation in strawberry fruit (*Fragaria ananassa*) (Miao et al., 2016). The impacts of the different light qualities on the anthocyanin content differed markedly between plants and tissues. Additionally, the effects of the red/blue combination were intermediated between blue and red lights, indicating that the functions among the blue and red lights on anthocyanin biosynthesis were redundant.

In this investigation, we discovered that there was an opposing trend between anthocyanin synthesis and photosynthesis: blue light promoted anthocyanin accumulation, but decreased photosynthesis; while photosynthesis was highest and the anthocyanin content lowest in white light when compared with the other light conditions. An increase in the accumulated anthocyanin causes the leaf color to turn from green to light-red as it decreases the chlorophyll content, and reduces the chlorophyll *a/b* ratios, resulting in reduced photosynthesis in broadleaf evergreen species (Hughes and Smith, 2007). In contrast, anthocyanin biosynthesis indirectly affects the circadian leaf starch metabolism and attenuates the sugar-promoted feedback resulting in the down-regulation of photosynthesis. The lower the observed photosynthesis rate decrease in red leaves with higher anthocyanin content than in the green leaves with lower anthocyanin accumulation (Landi et al., 2015; Piccolo et al., 2018). To some degree, the accumulation of anthocyanin inhibits photosynthesis (Piccolo et al., 2020). The blueberry leaves were treated with different light qualities and this affected the anthocyanin content which was significantly higher, but photosynthesis was lower under the blue and red/blue combination lights than under white and red lights. This is in accordance with previous reports, in which an increase in anthocyanin accumulation was accompanied by a reduction in photosynthesis (Murakami et al., 2008). Another explanation is that the abundant accumulation of anthocyanin in the leaves appeared to screen underlying photosynthetic tissues, increasing light saturation and compensation points, reducing the maximal photosynthetic assimilation rate (*A*_max_) (Nick et al., 2009). Notwithstanding the insights proposed by these investigations, the correlation and regulatory mechanism between anthocyanin accumulation and photosynthesis is yet to be defined under different light qualities, and future studies on blueberry leaves and other plant tissues will be required.

Recently, the integration of large-scale datasets derived from high-throughput functional genomics techniques has been successfully applied to study the functions of genes regulating tissue development, environmental responses, and plant metabolism (An et al., 2020; Raza et al., 2021). Comparative transcriptome analysis of bilberry (*V. myrtillus* L.), for example, under different light qualities (red and blue) has provided information about the gene transcript level and regulation related to anthocyanin biosynthesis (Samkumar et al., 2021). Our results showed that the majority of the DEGs in the flavonoid and anthocyanin biosynthesis pathways were up- and down-regulated under different light qualities in blueberry leaves using RNA-Seq. Anthocyanins are biosynthesized *via* a branch of the flavonoid pathway, and their synthesis is affected by the presence or absence of upstream genes. The anthocyanin biosynthesis pathway is relatively clear in plants, and related key structural genes, including *C4H*, *4CL*, *CHS, CHI*, *OMT*, *F3’H*, *F3’5H*, *DFR*, *LDOX*, *LAR*, *UFGT*, *UGT*, and *FLS*, which have been identified in the colored tissues of several plants (Gao et al., 2021). Blue light has been identified as a strong positive influence on anthocyanin biosynthesis in numerous fruit crops, for instance, in sweet cherries (Kokalj et al., 2019). Red light can also do it in similar ways (Zhang et al., 2018). Our results showed that blue and the red/blue light combination up-regulated most of the anthocyanin biosynthesis genes, including *CHS*, *CHI, F3H*, *DFR*, *LDOX*, *OMT*, and *UFGT*, in blueberry leaves, and this led to a higher accumulation of anthocyanin under both blue and red/blue light combination treatments when compared with white light. It should be noted that multiple unigenes were annotated as the same enzyme in this study. For example, three CHS-encoding genes and two DFR-encoding genes were identified. The main reason for this was that these unigenes belong to different selective splicing transcripts as well as a specific gene family (Yang et al., 2005).

The photoreceptor can induce the accumulation of anthocyanin by promoting the expression of anthocyanin biosynthesis genes. Red light, acting *via* phytochromes, stimulates PAL activity in the cotyledons and hypocotyls of tomato seedlings, and exposure to UV-B has been shown to stimulate PAL activity, thus, increasing anthocyanin content in rice, maize, and turnip plants (Brodenfeldt and Mohr, 1988; Reddy et al., 1994; Sharma et al., 1999). Phytochrome B has a specialized function in red light, whereas cryptochrome senses blue light to promote secondary metabolite synthesis and anthocyanin accumulation. Previous studies have reported that UV light regulates the synthesis of anthocyanins through different photoreceptors, including the UV-B receptor UV RESISTANCE LOCUS 8 (UVR8) (Brown et al., 2005; Christie et al., 2012; Giliberto et al., 2005). In this study, genes related to photoreceptors differed in their expression levels under different light qualities and subsequently played a potential role in anthocyanin accumulation. The DEGs related to phytochromes were highly expressed in response to red light, whereas cryptochrome-related DEGs were substantially expressed under blue light. These results indicate that the transcript levels of these photoreceptor genes were relatively high under their corresponding light quality, while the lowest expression level of the photoreceptor-related genes was observed under white light. Blue and red light may promote anthocyanin biosynthesis by inducing the expression of photoreceptor-related genes to stimulate the gene promoter activity involved in the anthocyanin biosynthesis pathway. Photosynthesis is an important physiological process in plants under light conditions, and the photosynthesis-related DEGs showed an opposite dynamic trend with DEGs related to anthocyanin synthesis in gene expression patterns, which was consistent with their corresponding physiological index content. This result may further confirm the above conjecture that anthocyanin accumulation may restrain photosynthesis to a certain extent in blueberry leaves.

In several cases, changes in the expression of genes related to anthocyanin biosynthesis correspond to changes in the content of anthocyanin derivatives in this pathway (Kyoungwon et al., 2016). Consistent with the transcriptome results, the level of metabolite accumulation in the anthocyanin biosynthesis pathway differed substantially under different light qualities. Anthocyanins are glycosides and acylglycosides of anthocyanidin aglycones that are biosynthesized through the flavonoid pathway *via* the phenylpropanoid pathway (Stushnoff et al., 2010; Jaakola, 2013). Among these metabolic pathways, cyanidin (Cy), delphinidin (Dp), pelargonidin (Pg), peonidin (Pn), petunidin (Pt), and malvidin (Mv) are the primary metabolic derivatives that play vital roles in anthocyanin pigmentation (Tahara, 2007). In this study, the accumulation levels of these derivatives differed under different light qualities; blue and red/blue could promote the synthesis of more metabolites than red and white lights could, which was consistent with the total anthocyanin content and leaf color under different light qualities. Among these derivatives, the accumulation of Cy, Pg, and Mv derivatives and Dp, Pn, and Pt derivatives were significantly higher under blue light and the red/blue light combination, respectively (**Figure 7**). It has been reported that the six anthocyanidins that generally occur in colored tissues are Cy, Pg, Mv, Dp, Pn, and Pt and that they play vital roles in anthocyanin pigmentation and biosynthesis. Additionally, in the correlation network between the transcriptome and metabolome of blue vs. white, Cy, Pg, and Mv were found to be closely associated with *LDOX*, *UFGT*, and *OMT*, respectively. Previous studies have reported that individual enzymes also have an impact on the overall stability of the biosynthesis pathway, as the silencing, over-expression, or heterologous expression of single enzyme genes often leads to substantial changes in the anthocyanin composition of the target tissue (Griesser et al., 2008; Han et al., 2010; Han et al., 2012). Therefore, as regulative downstream materials, the high expression of *LDOX*, *UFGT*, and *OMT* resulting in the abundant accumulation of Cy, Pg, and Mv under blue light in blueberry leaves. Similarly, in the red/blue vs. white correlation network, similar relationships were observed between Dp, Pn, and Pt and *DFR* and *OMT*. The expression levels of *DFR* and *OMT* were higher, when accompanied by the abundant synthesis of Dp, Pn, and Pt with the combination of the red and blue lights. This is consistent with a previous report by Kyoungwon et al. (2016), in which the Dp derivative was shown to positively correlate with the expression level of genes, including *DFR*, *CHS*, and *CHI*; consequently, the more significant up-regulation of these genes has a role in the more significant accumulation of the Dp derivative. *OMT*, a vital enzyme gene in the anthocyanin biosynthesis pathway, catalyzes the formation of *O*-methylated anthocyanins, such as Mv, Pn, and Pt (Koes et al., 2005). High levels of *OMT* expression can induce the accumulation of Mv, Pn, and Pt under blue and red/blue light. Thus, we concluded that blue and red/blue lights induce the activity of the DEGs associated with anthocyanin biosynthesis and consequently, this facilitates the accumulation of anthocyanins in blueberry leaves under blue and red/blue lights, respectively.

## Conclusion

**Figure 9:**
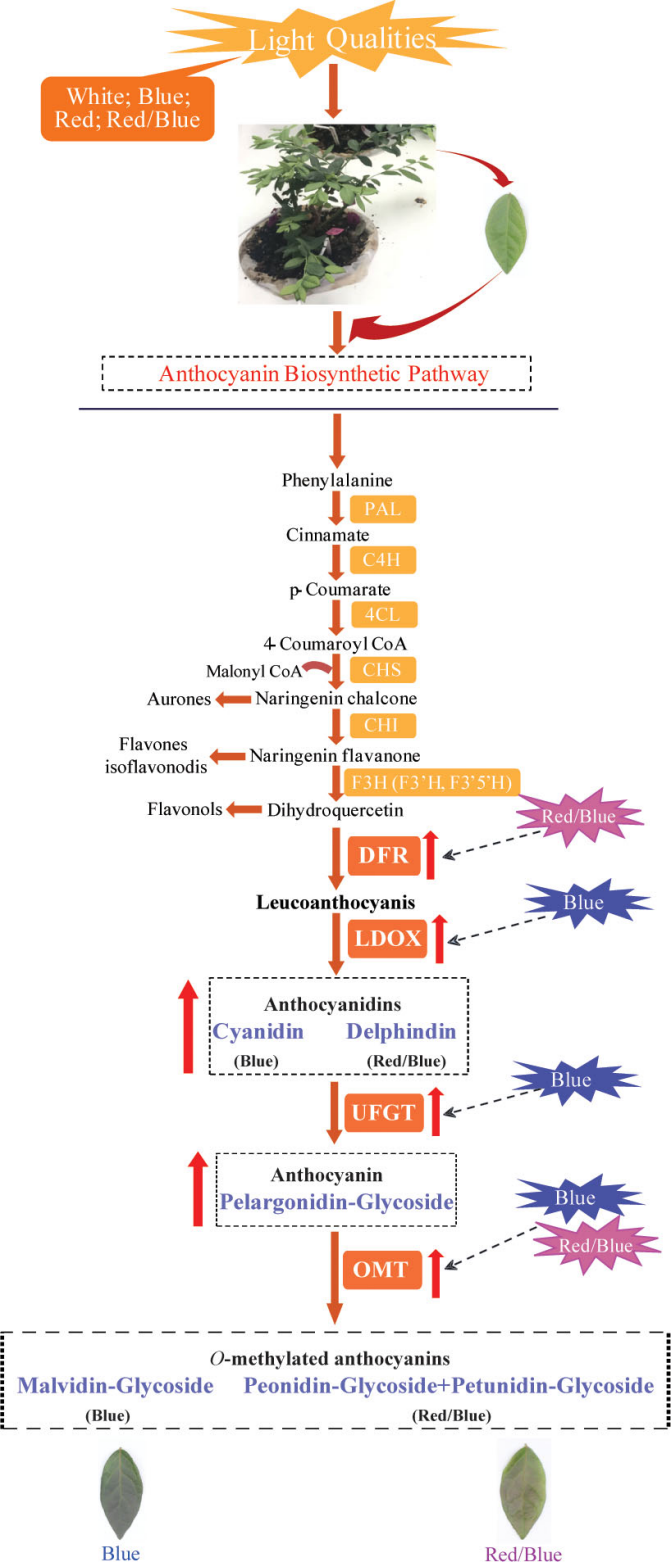
Proposed model of the molecular regulatory mechanism underlying anthocyanin biosynthesis in blueberry leaves in response to different light qualities. The red up-arrow indicates a significant increase; the dotted line arrow indicates a potential role for positive regulation.

In this study, blue and red/blue, and red lights were found to promote anthocyanin accumulation in blueberry leaves, and there was a redundant function that facilitated anthocyanin biosynthesis and accumulation between the blue and red lights. Additionally, correlation analysis of the changes in gene expression and metabolite levels suggested that blue light induced substantial *LDOX*, *UFGT*, and *OMT* expression to promote the accumulation of cyanidin, pelargonidin, and malvidin anthocyanidins; while the combination of red and blue light facilitated the up-regulation of *DFR* and *OMT*, leading to the accumulation of large amounts of downstream metabolites, including delphinidin, petunidin, and peonidin derivatives (**Figure 9**). Meanwhile, there was a sharp adverse dynamic trend between anthocyanin accumulation and photosynthesis in the blueberry leaves under different light quality treatments. The results obtained in this study provide new insights into the molecular mechanisms underlying anthocyanin biosynthesis and how the inter-correlation between anthocyanin accumulation and photosynthesis is regulated.

## Data availability statement

The data presented in the study are deposited in the NCBI repository with the link: https://www.ncbi.nlm.nih.gov/, accession number PRJNA880737.

## Author contributions

XZ and HA organized the entire project. JZ, HA, and SL performed the experiments and data analysis SL and BZ helped to review and modify the manuscript. XZ and HA edited the manuscript. All authors contributed to the article and approved the submitted version.
