# Supplementary figures and images for: Integrated transcriptome and metabolome analysis reveals the anthocyanin biosynthesis mechanisms in blueberry (Vaccinium corymbosum L.) leaves under different light qualities
Source: Front Plant Sci. 2022 Dec 8;13:1073332. doi: 10.3389/fpls.2022.1073332 (PMC9772006; doi:10.3389/fpls.2022.1073332)

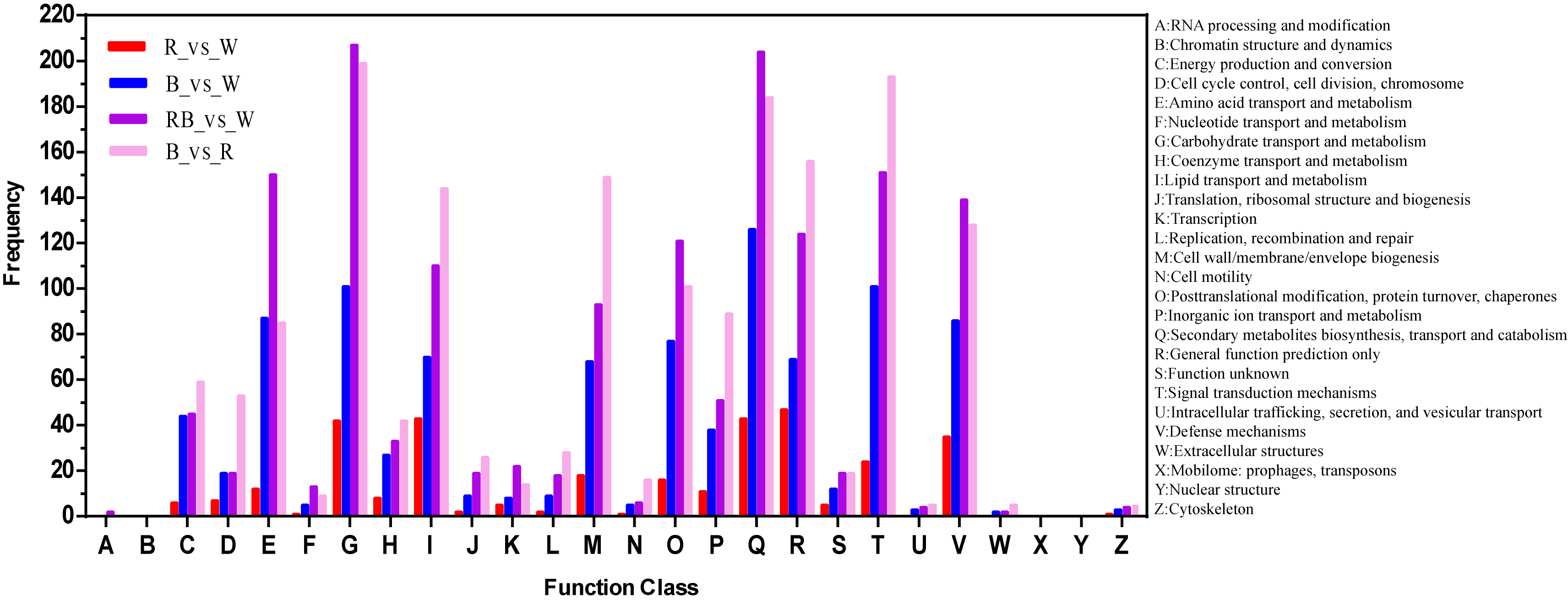

Supplement: Supplementary file 2 [file Image_1.tif]
